# Short-Term Effects of Meditation on Sustained Attention as Measured by fNIRS

**DOI:** 10.3390/brainsci10090608

**Published:** 2020-09-04

**Authors:** Meltem Izzetoglu, Patricia A. Shewokis, Kathryn Tsai, Phillip Dantoin, Kathryn Sparango, Katherine Min

**Affiliations:** 1 Electrical and Computer Engineering Department, Villanova University, Villanova, PA 19085, USA; ktsai2@villanova.edu (K.T.); pdantoin@villanova.edu (P.D.); ksparang@villanova.edu (K.S.); kmin2@villanova.edu (K.M.); 2 Nutrition Sciences Dept., College of Nursing and Health Professions; School of Biomedical Engineering Science and Health Systems, & School of Education, Drexel University, Philadelphia, PA 19104, USA; pas38@drexel.edu

**Keywords:** functional near infrared spectroscopy (fNIRS), meditation, Stroop task

## Abstract

Cognitive abilities such as attention, memory, processing time, perception, and reasoning can be augmented using some type of intervention. Within the broad range of conventional and unconventional intervention methods used in cognitive enhancement, meditation is one of those that is safe, widely practiced by many since ancient times, and has been shown to reduce stress and improve psychological health and cognitive functioning. Various neuroimaging studies using functional magnetic resonance imaging (fMRI) and electroencephalography (EEG) have shown functional and structural changes due to meditation in different types of meditation practices and on various groups of meditators. Recently, a few studies on meditation have used functional near infrared spectroscopy (fNIRS) to study the effects of meditation on cerebral hemodynamics. In this study, we examined the short-term effects of loving-kindness (LK) meditation on sustained attention using behavioral performance measures, physiological outcomes, and cognitive activity as measured by fNIRS in first-time meditators during Stroop color word task (SCWT) performance. Our results indicated that behavioral outcomes, assessed mainly on response time (RT) during SCWT performance, showed a significant decrease after meditation. As expected, physiological measures, primarily pulse pressure (PP) measured after meditation dropped significantly as compared to the before meditation measurement. For the hemodynamic measures of oxygenated-hemoglobin (HbO_2_), deoxygenated-hemoglobin (Hb), and total-hemoglobin (HbT), our findings show significant differences in SCWT performance before and after meditation. Our results suggest that LK meditation can result in improvements in cognitive, physiological, and behavioral outcomes of first-time meditators after a short-term session.

## 1. Introduction

Cognition refers to the mental processes such as attention, memory, language, visual processing, logic, and reasoning that are used to organize information [1,2]. Cognitive abilities or core capacities of the mental processes can be highly variable individually and developmentally regardless of the presence of any pathological conditions. It has been shown that cognitive abilities can be enhanced or augmented resulting in improved learning, focus, memory, reaction time, perception, and improved reasoning capacity using some type of intervention [2]. A widely accepted definition of enhancement is characterized as interventions in humans that aim to improve mental functioning beyond what is necessary to sustain or restore good health.

There is a broad range of interventions used in cognitive enhancement which are usually categorized as conventional or unconventional methods [1,2]. Conventional means of augmenting cognition are referred to as being of a more routine nature or have been practiced for many years, such as education and training, and hence well established and culturally accepted. In contrast, unconventional methods are relatively new, more experimental, and sometimes even controversial, where their safety and efficacy are not fully studied. Unconventional methods can involve invasive approaches such as stimulant drugs, gene therapies, and brain stimulation techniques or more noninvasive ones such as mental training involving yoga, martial arts, and meditation.

In a more recent review [3], seven essential dimensions of cognitive enhancement were explained: Mode of action, acceptance, cognitive domain, availability, personal factors, side effects, and temporal factors. In the same study, enhancement strategies were clustered into three major areas according to their main mode of action: (i) Biochemical interventions, e.g., nutrition, natural remedies, pharmaceuticals; (ii) physical interventions, e.g., implants and various forms of stimulations; and (iii) behavioral interventions, e.g., physical exercise, sleep, and meditation.

Of all these different classes or types of cognitive enhancement techniques, meditation is one of those that has been practiced globally by humanity since ancient periods in some form. There is highly anecdotal, as well as scientific and clinical evidence, as to its effects on stress relief, and relaxation, as well as the enhancement of attention, memory, and overall psychological health and cognitive functioning [1]. Studies have further demonstrated that meditation practice can cause structural changes in the brain that promote improvements in executive functions and attention regulation abilities and can even delay cognitive decline in the elderly and speed up learning in children [4]. In addition, meditation has been studied in various clinical populations and has been shown to lead to clinical outcomes in anxiety, depression, stress, and substance abuse problems [4,5,6,7,8].

A wide range of studies exist on meditation, and its effects on cognition in the literature in terms of the meditation practices have been studied (e.g., focused attention, open-monitoring, transcendental, and loving-kindness (LK) meditation), as well as the types of subjects enrolled (first time, novice, or expert meditators), the long-term (trait) vs. short-term (state) effects of meditation on cognition, the targeted cognitive domain (attention, memory, etc.), and the method of measuring cognitive enhancement (self-report, behavioral outcomes, brain activity obtained by neuroimaging techniques) [4]. Within this broad spectrum of studies, neuroimaging findings primarily based on electroencephalogram (EEG) and functional magnetic resonance imaging (fMRI) have helped in objectively clarifying the electrophysiological, structural, and functional underpinnings of the positive effects of meditation practice [5,6,7,8]. EEG studies on meditation, conducted for almost 50 years, have shown increases in theta and alpha band powers with an overall frequency slowing (reduction in band frequencies) and increased coherence [8]. EEG studies suggested specific differences in EEG oscillations among different types of meditation practices. Meditation studies using event related potentials (ERP) have shown changes in amplitude and latency in some components and practices, which suggested increased attentional resources and stimulus processing speed or efficiency [8,9]. However, the lack of consistency in the meditation studies with EEG and limited information on the location of observed neural activation differences have somewhat limited the conclusiveness of the findings.

Neuroimaging studies using fMRI have overcome some of the limitations of EEG studies and have provided valuable information on spatial localization of brain activations during meditation [10,11]. Those findings indicated increased regional cerebral blood flow during meditation, specifically in frontal and prefrontal areas, which suggest increased attentional demand of meditative tasks and alterations in self-experience [8]. A more recent meta-analysis of fMRI results, as discussed by the authors of [5], suggested that meditation practice can result in functional and structural brain modifications, specifically in areas involved in self-referential processes, such as self-awareness and self-regulation as well as in areas involved in attention, executive functions, and memory formations such as in prefrontal cortex (PFC). However, fMRI can pose some challenges in meditation studies such as a loud, restrictive environment, laying in supine position, low temporal resolution, high sensitivity to participant’s motion, and relatively high cost [12]. Some of these challenges can be overcome by a relatively new brain imaging modality, namely functional near infrared spectroscopy (fNIRS) technique.

fNIRS is an optics-based brain imaging modality which can measure relative changes in oxygenated (HbO_2_) and deoxygenated (Hb) hemoglobin using light in the near infrared range (650–950 nm). Due to its noninvasive, safe, portable, easy-to-use, and low-cost characteristics and its relative immunity to movement artifacts, it has been used in cognitive activity monitoring in many studies that required ecological validity in the field, as well as in clinics [13,14,15] Very recently, fNIRS technology was applied in meditation research and the study of higher states of consciousness induced by psychedelics [12,16,17,18,19]. Within these limited number of fNIRS studies on meditation, the authors of [17] measured cerebral blood flow using fNIRS on a veteran and a beginner practitioner during Taichi-quan twice, and they observed increased blood flow in the PFC regions in both subjects. The authors of [18] demonstrated that Hb decreased significantly with a subsequent increase in HbO_2_ in the left PFC during Qigong meditation in practitioners as compared to nonpractitioners. The authors of [16] used fNIRS on meditation experts and a control group during mindfulness meditation and resting conditions. The sound of a meditation bowl was used to find group differences in the hemodynamic responses in the auditory system and adjacent cortical areas where spatial activation differences were found in expert meditators and controls under mindfulness and resting states [16]. In [12], Deepeshwar et al. used fNIRS to assess differences in brain activations of expert meditators during the performance of a sustained attention task, namely the Stroop color-word task (SCWT), before and after effortless meditation (mental chanting of ‘OM’ and random thinking sessions). It was found that during meditation, there was an increase in HbO_2_ and total hemoglobin (HbT = HbO_2_ + Hb), with reduced Hb concentration over the right PFC. During SCWT, the mean reaction time (RT) was shorter and HbT was reduced after mantra meditation, indicating improved neural efficiency and enhanced performance in a sustained attention task on expert meditators.

To our knowledge, a limited number of meditation studies exist that have studied the short-term effects of different types of meditation on expert meditators using fNIRS. Prior EEG and fMRI studies have also shown that meditation can augment cognition by comparing expert meditators to controls. Although there is a paucity of research on novice meditators, the study of cognitive and behavioral effects of meditation on completely inexperienced individuals in meditation can provide important information and positive implications to our understanding on the impact to cognitive enhancement for this group of performers. For example, if meditation is found to improve cognition in first-time meditators and in the short-term, it can signify the utility of meditation as an acute cognitive enhancement technique which can be used volitionally as needed.

The aim of this study was to examine the short-term effects of meditation on sustained attention, behavioral performances, and physiological outcomes of first-time meditators during SCWT. The study involved healthy young adults (*n* = 20) who never had a prior meditation experience. Participants performed three conditions, SCWT once before and once after ~22 min of guided, LK meditation. Throughout the entire procedure, the participants’ brain activity in the dorsolateral prefrontal cortex (DLPFC) was monitored using fNIRS. Behavioral responses to SCWT in terms of response accuracy (RA) and RT were also collected. In addition, physiological changes, such as systolic blood pressure (SBP), diastolic blood pressure (DBP), pulse pressure (PP = SBP − DBP) and mean arterial pressure (MAP = (SBP + 2DBP)/3), and heart rate (HR), were also collected in the beginning and end of the overall procedure and right before and right after meditation task. After processing of the fNIRS recordings, three hemodynamic measures were derived for each condition, channel, and participant, namely mean HbO_2_, mean Hb, and mean HbT. We predicted that for the behavioral and physiological measures, there would be an improvement in the outcomes after the LK meditation. For the hemodynamic measures, we validated the impact of meditation with fNIRS, where we hypothesized that the mean fNIRS measures would increase during the LK meditation. In addition, we hypothesized that there would be changes in the fNIRS measures in the Stroop task before and after meditation.

## 2. Materials and Methods

### 2.1. Participants

A total of *n* = 21 undergraduate students ranging from 18 to 21 years old (mean age 20.5 ± 0.96; 10 males, 11 females) were recruited from Villanova University student body through flyers posted on university campus and sent out via email to general student mailing lists. All participants were native English speakers, right-handed, vision correctable to 20/20, and did not have color-blindness or any neurological disorders (e.g., strokes, concussions, or other brain injuries). The study was approved by the Institutional Review Board (IRB) at Villanova University (IRB-FY2019-230) and the participants signed the informed consent before starting the test procedures. We based our sample size on previous studies conducted by the authors of [12] and [20], which had *n* = 22 and *n* = 20 participants, respectively. We lost one participant’s fNIRS data because of extreme responses yielding a total sample size of *n* = 20. For the physiological measures, a subsample was used given various equipment concerns (*n* = 10).

### 2.2. Procedure

**Figure 1:**
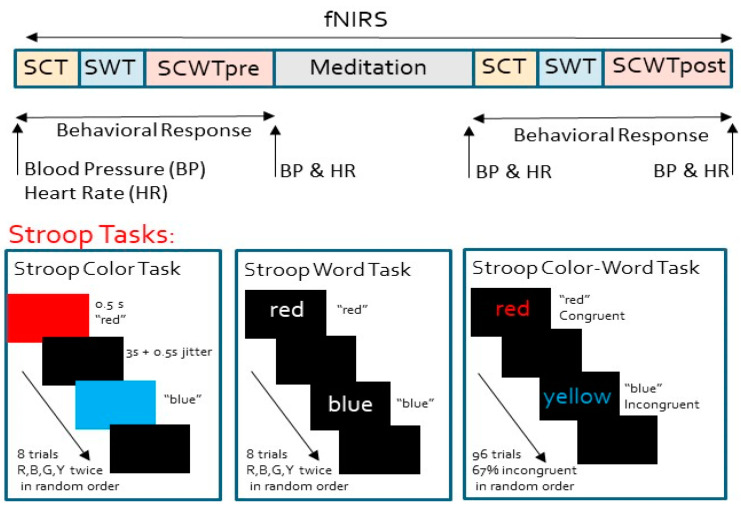
Overall task protocol and procedures.

Overall task procedures are shown in the block diagram in Figure 1. The study started with the regular consenting process of obtaining written informed consent and enrolling the participants in the study. All procedures were performed while participants were sitting in a comfortable chair in quiet room in front of a computer. After the placement of the fNIRS sensor on the participant’s forehead for continuous PFC activity monitoring and the blood pressure sensor was placed o to the participant’s right arm for intermittent physiological data recordings, the task protocol started. The protocol involved sequential performance of first a Stroop color task (SCT) and then a Stroop word task (SWT) for baseline cognitive activity monitoring which took ~1 min, the first administration of ~6 min of SCWT pre-meditation (SCWT-pre), LK meditation of ~22 min, second administration of another baseline SCT and SWT for a total of ~1 min, and finally, ~6 min of SCWT post-meditation (SCWT-post). During the overall task procedure, fNIRS data and behavioral responses to SCT, SWT, and SCWT pre- and post-meditation were collected continuously. Physiological data in terms of BP and HR were collected intermittently in the beginning and end of the test, as well as in the beginning and end of the meditation procedure. Overall, the procedure lasted to an hour per subject.

#### 2.2.1. Loving-Kindness Meditation

Since the participants in this study were all first-time meditators, we selected to use a guided meditation of the Loving-Kindness type. In order to keep the meditation experience the same for each participant, each participant listened to a prerecorded, 22-min, 10-s Loving-Kindness podcast by psychologist and meditation teacher Tara Brach, PhD [21]. This meditation type and podcast was chosen for our study in consultation with Campus Ministry, which leads on-campus meditation services at Villanova University.

#### 2.2.2. Stroop Procedure

We adapted a modified version of the standard Stroop procedures [10,11,22,23], which included three tasks: SCT and SWT as baseline conditions and a modified form of SCWT [9,20,24], administered the same order pre- and post-meditation. In SCT, participants were asked to press the ‘r’, ‘b’, ‘y’, or ‘g’ keys on the computer keyboard for each of the color stimuli, presented as red, blue, yellow, and green color blocks on the computer screen, respectively. There were two repetitions of each color, adding up to eight trials in this task. In SWT, participants were asked to respond by pressing the ‘r’, ‘b’, ‘y’, or ‘g’, keys on the keyboard for the words ‘red’, ‘blue’, ‘yellow’, and ‘green’ printed in white on a black computer screen, respectively. Again, two sets of each color word were used, totaling eight trials for this task. During the SCWT, the stimuli were the same four color words used in SWT (‘red’, ‘blue’, ‘yellow’, and ‘green’), and were written in one of these four colors on a black background, so that the stimulus was either matching with the color and meaning of the word (the congruent stimuli) or the color was not matching the meaning of the word (incongruent stimuli). The participant was asked to respond to the ink color of the printed word by pressing ‘r’, ‘b’, ‘y’, or ‘g’ keys on the keyboard and not the meaning of the word (i.e., respond with ‘r’ to the word blue written in red ink). In our application, 33.3% of the trials were congruent stimuli and the remaining 66.7% of the trials were incongruent stimuli [24]. Each SCWT consisted of 4 blocks of 24 trials (total of 96 trials), where each block contained 8 congruent trials (2 trials of 4 ink colors matching the word meaning), and 16 incongruent trials (all possible incongruent combinations of 4 ink colors with color words, 1 trial each). With this arrangement, 1/3 of the trials were congruent stimuli and the remaining 2/3 of the trials were incongruent stimuli [25]. In each SCWT block, SCT and SWT, the stimuli were presented in random order. In these tasks, each stimulus stayed on screen for 0.5 s with an interstimulus interval of 3 s plus a random jitter of up to 0.5 s where a black screen was shown, and the participant’s response was recorded.

### 2.3. Data Acquisition

#### 2.3.1. Behavioral Recordings

The overall procedures were presented using Neuropsychological Assessment Research (NAR) stimulus presentation program developed at Drexel University (Hasan Ayaz). The NAR program was readily synced with the fNIRS device where each task’s start and end times were recorded as fNIRS markers for data epoch extractions and further analysis. In a separate text file, the NAR program also recorded the timing of each stimulus, screen presentation, and participant’s behavioral response (keys that were pressed), as well as their timing for response accuracy and behavioral response time extraction.

#### 2.3.2. Physiological Measurements

For the intermittent BP and HR measurements, we used a commercially available device OMRON HEM-712C Automatic Blood Pressure Monitor by OMRON Healthcare Inc., Bannockburn, IL, USA. The pressure cuff of the device was placed on the right upper arm of the participants in the beginning of the protocol when the first BP and HR measurements were collected. The deflated pressure cuff stayed on during the course of the procedure until the protocol was finalized where BP and HR measurements were collected intermittently once before the meditation, once after it, and once when the overall task was finalized. These physiological recordings were entered to a separate secured data sheet by the experimenters for each corresponding participant.

#### 2.3.3. Hemodynamic Response Measurements via fNIRS

**Figure 2:**
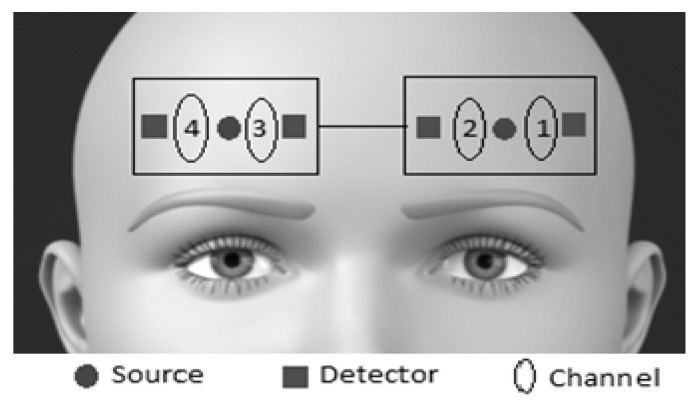
fNIRS sensor placement on the forehead with corresponding channel recording locations.

Hemodynamic responses from the DLPFC were collected using fNIR Imager, 1200 W (fNIR Devices, LLC., Photomac, MD, USA). The fNIRS system consisted of two identical sensors that were placed one on the left and the other on the right side of the participants’ forehead using standard procedures (Figure 2), a wireless control box for data acquisition, and a computer for data collection and storage. The identical sensors were built on a flexible circuit board which was covered with a silicone material for comfort, sealing, durability, and hygiene. Each fNIRS sensor consisted of one LED light source in the middle and two photodetectors on each side of it, which were arranged in a linear form with a source-detector separation of 2.5 cm. The light sources on the sensor (Epitex Inc., Kyoto, Japan, type L4 × 730/4 × 805/4 × 850-40Q96-I) contained three built-in LEDs having peak wavelengths at 730 nm, 805 nm, and 850 nm, with an overall outer diameter of 9.2 mm ± 0.2 mm. The photodetectors (Bur Brown, type OPT101) were monolithic photodiodes with a single-supply transimpedance amplifier. The system could collect data at a sampling rate of ~4.43 Hz.

Since the fNIRS sensor is flexible, the components can move and adapt to the various contours of the participants’ foreheads, allowing the sensor elements to maintain an orthogonal orientation to the skin surface, improving light coupling efficiency and signal strength. The standard sensor placement procedure was followed where the middle point where the two identical sensors were connected to each other was placed on the forehead so that the horizontal symmetry axis central (y-axis) coincides with symmetry axis of the head, (i.e., in between the eyes). On the vertical axis, the sensors were positioned right above the eyebrows. With this configuration, fNIRS system collected data from leftmost, left-middle, right-middle, and rightmost forehead locations in its channels 1, 2, 3, and 4, respectively, as shown in Figure 1. The fNIRS data were collected using the Cognitive Optical Brain Imaging (COBI) studio data collection software of the fNIRS system running on a Windows 10 laptop computer, where the Stroop Test was also administered using the synched stimulus presentation software, NAR program.

### 2.4. Data Analysis and Statistics

Out of the 21 participant recordings, one participant’s fNIRS data was not recorded for the full procedure and was excluded from further analysis. Hence, fNIRS analysis was based on available *n* = 20 participant data. Furthermore, the physiological data, BP, and HR were not collected in 10 participants. Therefore, the analysis on physiological data was based on the number of participants where BP and HR recordings existed.

#### 2.4.1. Behavioral Performance and Physiological Data Analysis

Within the Stroop tasks, the percentage of the correct responses were used for response accuracy (RA), and the average value of the time it took for the participants to correctly respond to the stimuli was taken as the behavioral response time (RT). Hence, for the RT incorrect responses, the times were not used. The available physiological recordings, MAP, PP, and HR were matched with the corresponding participant’s RA and RT values and fNIRS markers.

#### 2.4.2. fNIRS Data Processing

Initially, data from each of the four fNIRS channels were visually inspected for saturation or dark current conditions or for extreme noise, which could happen due to incorrect sensor placement, and the identified ones were eliminated from further analysis (12.5% of the data eliminated, mainly on the right most channel 4 location). Then, we applied wavelet denoising to the remaining raw intensity measurements at 730-nm and 850-nm wavelengths for spiky noise suppression [25]. Changes in oxygenated hemoglobin (HbO_2_) and deoxygenated hemoglobin (Hb) were calculated from those artifact-removed raw intensity measurements relative to the resting baseline collected in the beginning of the task using modified Beer–Lambert law as previously described [13,26]. To remove possible baseline shifts and to suppress physiological artifacts such as respiration and Mayer waves, we first applied Spline filtering [27] followed by a finite impulse response low-pass filter with cut-off frequency at 0.08 Hz [26].

In this study, the procedure was designed where tasks followed each other continuously with no resting periods in between. Since the hemodynamic response is a slow process, some hemodynamic effects from a preceding task could have been carried over to the following task. Furthermore, the meditation podcast used in this study had a silent period at the very end of it without guidance to meditation. In order to eliminate such possible artifacts, the data epochs for SCWT-pre, SCWT-post, and meditation tasks, the ones primarily considered in further statistical comparisons in this study, were selected as the recording region between 15 s after the start of the task and 15 s before the end of the task to be consistent. In addition, to further eliminate any cumulative effect due to the continuous application of different tasks, relative changes in hemodynamic responses during SCWT-pre and SCWT-post conditions were obtained in relation to the prior SCT and SWT baseline data using their overall mean value in a baseline correction procedure (subtracting the mean of the Stroop baselines from the SCWT task period). To be consistent, change in meditation data epoch was calculated relative to the first 15 s data interval in the beginning of the meditation task by applying similar baseline correction process. After selecting the task epoch region and applying the baseline correction on them, in order to reduce dimensionality, we extracted the average value of HbO_2_, Hb and HbT (HbO_2_ + Hb) recordings for each SCWT-pre, SCWT-post, and meditation task, channel, and subject separately, and used it as features in further statistical comparisons. We calculated laterality indices as described by the authors of [28] for continuous wave fNIRS measurements. This calculation involved the maximum values, and we used the maximum value of channels 1 and 2 (for the left PFC; max HbO_2_) and of channels 3 and 4 (for the right PFC; max HbO_2_). For both HbO_2_ and Hb, the laterality index (LI) was calculated using Equation (1). The LI ranged from −1 to +1. A positive LI indicated that the person was left lateralized, and a negative LI indicated that the person was right lateralized.

(1) $$LI=\;\frac{\left(\max HbO_{2}\_L\right)-\left(\left(\max HbO_{2}\_R\right)\right)}{\left(\left(\max HbO_{2}\_L\right)+\;\left(\max HbO_{2}\_R\right)\right)}$$

#### 2.4.3. Statistical Analysis

There were three sets of dependent measures assessed in this study: Behavioral (RT–msec, RA–percent correct), physiological (MAP–mmHg, PP–mmHg, HR–bpm), and fNIRS (mean HbO_2_, mean Hb, mean HbT). For the behavioral and physiological measures, there was one within the subject variable: Condition with two levels (pre-meditation; post-meditation), resulting in paired *t*-tests calculated for these measures. Cohen’s d_z_ effect size was calculated and used to aid in the interpretation of the effects [29]. Prior to the primary fNIRS assessments, we created multiple measures to assess the effectiveness of meditation on performance. The impact of meditation on HbO_2_, Hb, HbT, MAP, PP, and HR were calculated by subtracting the SCWT-pre scores for each variable from the meditation score. Then, we calculated a series of regression models where the effect of meditation (e.g., MedeffHb) and SCWT-pre (RA) were predictors in linear regression models to predict the behavioral SCWT-post (RA) scores. We calculated these meditation effects given the preliminary nature of the study and the lack of a control group. For the primary fNIRS measures, there were two within the subject factors: Condition with three levels (SCWT-pre, meditation, SCWT-post) and Channels with four levels (1, 2, 3, 4). Channels were analyzed separately with one-way repeated measures analysis of variance (ANOVA) on Condition for each fNIRS feature. Violations of sphericity were adjusted with Greenhouse–Geisser (G–G) corrections. Follow-up polynomial contrasts were calculated to detect linear or quadratic trends. To identify the meaningfulness of significant omnibus F-tests, partial eta-squared (η^2^_partial_) effect sizes are reported representing the proportion of variance in the dependent measure explained by the model. A tertiary set of analyses were conducted to determine if there were lateralization differences and the impact of mediation on lateralization. Two sets of analyses were conducted. First, to address if there was any laterality present in each of the phases (pre-meditation, meditation, and post-meditation), one-sample *t*-tests with the comparison value of zero (0) were conducted. Assumptions were tested and met. Then, one-way repeated measures ANOVAs were conducted to determine if there were any differences in lateralization across the three phases. If there was a significant omnibus F, Bonferroni corrected post-hoc comparisons were calculated and polynomial contrasts for linear and quadratic trends were conducted. As well, Cohen’s d_z_ effect sizes were calculated for all tests conducted [29]. A significance criterion of α = 0.05 was used for all tests. To control for Type I error inflation, there were two families of tests, behavioral and physiological measures, and fNIRS measures, which were adjusted by a Benjamini–Hochberg False Discovery Rate (FDR) adjustment [30]. Analyses were conducted in R (R Core Team, 2019) and figures were produced using the packages ggpubr and ggplot2 [31].

## 3. Results

We hypothesized that, after meditation, there would be improvements in the behavioral (RA and RT) and physiological (MAP, PP, and HR) measures. For the pre-meditation vs. post-meditation Conditions, the descriptive statistics, confidence intervals, and Cohen’s d_z_ effect sizes of the behavioral and physiological measures are reported in Table 1.

### 3.1. Behavioral Outcomes

**Figure 3:**
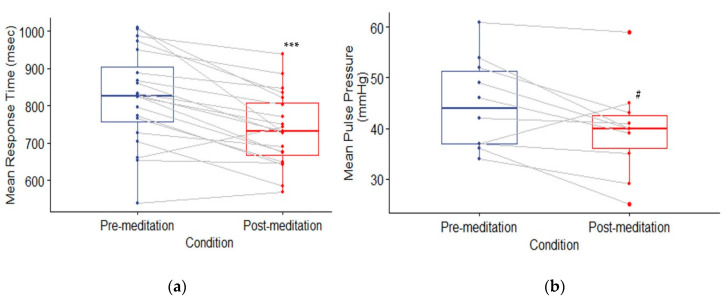
Comparisons of Pre-meditation (blue) to Post-meditation (red) conditions for (**a**) behavioral and (**b**) physiological outcomes. Mean response time (RT) in ms is the behavioral outcome (*n* = 20), while the mean pulse pressure (PP) in millimeters of mercury (mmHg) is the physiological outcome (*n* = 10). Grey lines represent each subject’s pre-meditation and post-meditation responses. *** (*p_FDR_* < 0.001), # (*p_FDR_* = 0.07).

All participants’ behavioral outcomes (*n* = 20) based on RA (percent correct answers) and average RT (ms) on correct responses during the performance of the Stroop task before and after meditation were compared using separate paired *t*-tests. For RA, there was not a significant increase in accuracy comparing pre-mediation to post-meditation scores (*t*(19) = 1.426, *p_FDR_* = 0.273). The lack of an increase in RA may have been due to a ceiling effect, since RA was initially very high (>90%) before meditation (Table 1). However, there was a significant decrease of 83.8 ms in RT after meditation (*t*(19) = 4.997, *p_FDR_* < 0.001), which corresponded to a 10.2% improvement (see Table 1; d_z_ had a large effect) in behavioral performance of the Stroop task post meditation (Figure 3a). This result, we suggest, is a meaningful and supports our hypothesis of the improvement in behavioral performance after meditation.

### 3.2. Physiological Measures

A subsample of participants (*n* = 10) completed physiological measures. To assess the hypothesis that physiological measures, based on blood pressure (MAP and PP in mmHg) and heart rate (HR in bpm), would improve after meditation, separate paired *t*-tests were conducted on pre- and post-meditation measurements. For PP, there was a trend reduction after meditation (*t*(9) = 2.42, *p_FDRj_ =* 0.070) (Figure 3b), which is in line with prior findings on the effects of meditation on blood pressure response even on first time meditators [32]. None of the other results for MAP (*t*(9) = −0.167, *p_FDR_* = 0.871) and HR (*t*(9) = 1.317, *p_FDR_* = 0.275) were significant or trended in the appropriate direction. Although the findings were limited to PP at an acute reduction of 5.2 mmHg, the findings need to be interpreted with caution, given that we tested a subsample. It should also be noted that there was low statistical power along with higher variability, with effect sizes ranging in the sample. Future work could benefit from increased sample size and heart rate variability measures to assess the effectiveness of meditation and stress reduction [33].

### 3.3. Hemodynamic Responses

Descriptive values and inferential statistical comparisons were made using the selected feature, the average value for HbO_2_, Hb, and HbT (in μMol), for all channels separately using all available subject data (*n* = 20; see Table 2). To validate the impact of meditation with fNIRS, we hypothesized that the hemodynamic responses would increase during meditation relative to the Stroop tests before and after meditation. Prior to testing this hypothesis, we assessed the effect of meditation on predicting behavioral responses using linear regression equations. These tests are represented in Table 3. These analyses suggest a strong meditation effect, reflected in adjusted multiple correlation coefficients ranging between 60.7% to 76.6% to explain a substantial proportion of variation in the SCWT-post-meditation behavioral scores of RA and RT. We felt confident moving forward with our primary analyses.

**Figure 4:**
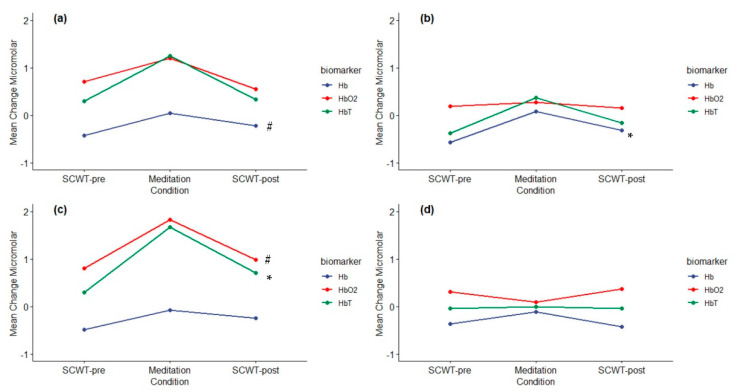
Mean fNIRS hemodynamic responses across the Conditions representing the polynomial contrasts quadratic trend analyses for each channel with (**a**) (Channel 1; N = 20), (**b**) (Channel 2 t; *n* = 20), (**c**) (Channel 3; *n* = 19), and (**d**) (Channel 4; *n* = 15). fNIRS biomarker measures were represented by HbO_2_-oxygenated hemoglobin (red line); Hb—deoxygenated hemoglobin (blue line); and HbT—total hemoglobin (green line). There were two significant quadratic trends denoted by symbol and color to the right of the trend line * (*p* < 0.05), * (*p* < 0.05) and there were two trends that approached significance, ^#^ (*p* > 0.051–0.100).

Separate one-way repeated measures ANOVAs were calculated across Conditions for each hemodynamic measure (i.e., HbO_2_, Hb and HbT) and each Channel separately. Only the significant or approaching significant results are reported. For HbO_2_, Channel 3 approached significance across Conditions (F_(2,36)_ = 2.705, *p* = 0.080, η^2^_partial_ = 0.131). The polynomial contrast resulted in a quadratic trend that also approached significance (F_(1,18)_ = 4.214, *p* = 0.055), which is depicted in bottom panel of Figure 4. This trend is in the appropriate direction, although the high variability for the measures across the conditions reflects an increased Type II error and low statistical power. Our results suggest that there was increase in HbO_2_ at ~22 min of LK meditation session in first-time meditators with a decrease in systolic blood pressure similar to prior neuroimaging findings on expert meditators [8,12,16].

For Hb, there were two findings. Channel 1 resulted in a significant difference across the Conditions (F_(1.399, 26.574)_ = 4.170 (G-G), *p* = 0.039, η^2^_partial_ = 0.180) with the polynomial contrast denoting an approach to significance for a quadratic trend (F_(1,19)_ = 4.213, *p* = 0.054) (see top panel of Figure 4). A significant difference across Conditions was also noted for Channel 2 (F_(2,38)_ = 4.792, *p* = 0.04, η^2^_partial_ = 0.201), with the polynomial quadratic trend similarly significant (F_(1,19)_ = 6.706, *p* = 0.018) (see middle panel of Figure 4). It is important to note a distinction between our Hb results and those described by the authors of [12]. Our sample had individuals inexperienced with meditation, while the sample in Reference [12] had individuals with at least 12 months experience in meditation on the Sanskrit syllable “OM” that were recruited from a university that focused on yoga practices. As well, the magnitude of the change in Hb values after meditation were higher in our sample relative to Reference [12]. Inspection of the standard deviation values for Hb across the Conditions (see Table 2), clearly shows that variability was reduced for Hb across Conditions while HbO_2_ and HbT had higher variability. It is possible that the inexperience of the participants and the limited channels may have contributed to the reduced Hb values as well.

Similar to HbO_2_, HbT resulted in significant differences in Channel 3 (F_(2,36)_ = 3.79, *p* = 0.033, η^2^_partial_ = 0.173) with a significant quadratic trend (F_(1,1)_ = 4.959, *p* = 0.039). The quadratic trend is illustrated in Figure 4 and provides support for the validation of an acute meditation training impact on hemodynamic responses. This finding is consistent with that described by the authors of [12], which illustrates support for the role of the DLPFC in cognitive control.

To determine if there were lateralization differences for the HbO_2_ and Hb laterality indices on the Stroop tests before and after meditation, one-sample *t*-tests were conducted on the measures compared to an alternative hypothesis that the mean is different from zero (0). Results of the analyses are reported in Table 4. The laterality index for HbO_2_ after meditation approached significance (*p* = 0.072), while the laterality index for the Stroop tests post-meditation was significantly different than 0 (*p* = 0.002, LI mean difference 0.522, d = 0.837), suggesting a reliable, large left lateralized effect for Hb. To determine the effect of meditation on lateralization, one-way repeated measures ANOVAs were conducted to determine if there were any differences in lateralization across the three phases for the HbO_2_ and Hb laterality indices. The laterality index for HbO_2_ (F_(2,36)_ = 0.466, *p* = 0.0631, η^2^_partial_ = 0.025) showed no differences in lateralization across the SCWT-pre meditation and SCWT-post for HbO_2_. For Hb, there was a significant omnibus effect (F_(2,36)_ = 8.179, *p* = 0.001, *p*_adj_ = 0.002, η^2^_partial_ = 0.31), with the polynomial linear trend similarly significant (F_(1,36)_ =3.385, *p* < 0.001, *p*_adj_ < 0.001). In addition, FDR adjusted pairwise contrasts yielded significant differences between SCWT-pre-meditation and SCWT-post-meditation (*p*_FDR_ = 0.003, d_z_ = −0.822; see Table 4 for the mean laterality scores) and meditation and SCWT-post-meditation (*p*_FDR_ = 0.005, d_z_ = −0.783). These significant findings represent large, reliable effects that illustrate that meditation reflects a move toward left frontal lateralization.

**Figure 5:**
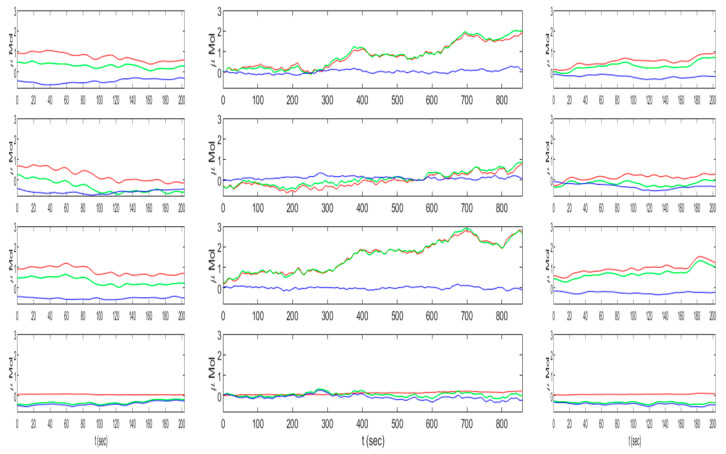
Average HbO_2_ (red), Hb (blue), and HbT (green) data epochs for SCWT-pre meditation and SCWT-post tasks in columns left, middle, and right for all subjects and channels 1, 2, 3, and 4 in rows from top to bottom, respectively.

In addition to the selected feature statistical outcomes, in order to provide more information on signal trends during different task conditions, all subject average HbO_2_ (red), Hb (blue), and HbT (green) data epochs in SCWT-pre, meditation, and SCWT-post task conditions (in columns from left to right) for all channels (1 through 4 from top to bottom) are shown in Figure 5. During meditation, HbO_2_ increased constantly (specifically in channels 1 and 3), which is in line to prior meditation study outcomes [12,17,18], suggesting an increase in focus during meditation. Both during SCWT-pre and SCWT-post, HbO_2_ and Hb showed reverse patterns in line with prior fNIRS studies [14,15]. The average value of HbO_2_ was similar during SCWT-pre as compared to SCWT-post. However, there was a significant increase in the average value of Hb from SCWT-pre to SCWT-post. Even though the average value in HbO_2_ was not significantly different between pre-meditation and post-meditation Stroop tasks, there was a clear difference in signal trends. HbO_2_ had a decreasing trend during the time course of SCWT-pre. Conversely, in SCWT-post, HbO_2_ had an increasing trend. Taking into account the reduction in response times with similar accuracy and hence improvement in behavioral outcomes after meditation, these HbO_2_ signal trends may suggest that participants were able to stay more focused during Stroop performance after meditation, which was not the case with Stroop performance prior to meditation.

## 4. Discussion

In this study, we investigated the short-term effects of LK meditation on sustained attention on the performance of SCWT in first-time meditators. We studied the behavioral performance outcomes, specifically the RT, to correct answers for congruent and incongruent trials in SCWT of the participants. The behavioral results indicated that, even though the percent accuracy in SCWT trials (>90% before and after meditation) did not improve, which was possibly due to a ceiling effect, RT during SCWT performance showed a significant decrease (83.8 ms) after meditation, suggesting a performance improvement. As the RT in SCWT was decreased after meditation as compared to before meditation, there was a corresponding increase in hemodynamic responses in the DLPFC region, suggesting improved attentional focus and behavioral performance even in first-time meditators, similar to prior findings on expert meditators [12]. Physiological measures, specifically SBP, decreased significantly (3.8 mmHg) after meditation as compared to before meditation, indicating reduced stress due to meditation practice, which is in alignment with prior literature. Similar to prior neuroimaging results, our results showed increased HbO_2_ and HbT during meditation due to increased attentional focus. In addition, measures of HbO_2_, Hb, and HbT on different channels on the left or right hemispheres also showed significant differences in SCWT pre- and post-meditation, suggesting that meditation may have resulted in changes in functional brain activity during the performance of a sustained attention task. Overall, our results suggest that LK meditation resulted in short-term improvements in cognitive, physiological, and behavioral outcomes of first-time meditators.

There were several limitations of the study. Only first-time meditators were tested in the study before and after a meditation session. It is important to include another testing session where the meditation task would be replaced with only a resting period that would be carried out on the same individuals. This methodological control could then identify if the changes observed in Stroop performance after meditation were due to the positive effects of meditation or they were just due to practice. In addition to a control task, experienced meditators, different types of meditation, and the effects of meditation in different components of attention and memory can further be studied. In SCWT, we followed a very conservative approach, applying congruent-to-incongruent ratio selection that was shown to generate the least Stroop effect [24]. Though we still were able to see the Stroop effect even with this conservative approach in our study, in the future, the congruent-to-incongruent ratio in the Stroop task can be reversed to generate a more distinct Stroop effect, which can provide a better indication on the effects of meditation in sustained attention. Finally, we used a wireless fNIRS system with only four channels. In the future, an fNIRS system with more channels that can cover the full forehead can be used, which can better identify location wise differences in DLPFC activations. Even though the current wireless four-channel fNIRS system used in this study offered advantages in terms of comfort and minimal disturbance allowing better focus in meditation and attention related tasks, it did not include short source-detector channels to account for the influence of extracerebral signals (e.g., blood flow in skin or connective tissue) as mentioned by the authors of [34]. We used well-accepted noise removal methods, which may have also eliminated the systemic artifacts from the skin. However, the utilization of short-source detector separation recordings can further be studied in the future for the elimination of possible other effects from the superficial layers.

## Figures and Tables

**Table 1:** Descriptive statistics for the behavioral ^1^ (response accuracy (RA), response time (RT)), and physiological ^2^ (mean arterial pressure (MAP), pulse pressure (PP), and heart rate (HR)) measures for the pre-meditation and post-meditation conditions.

Dependent Variable	Condition	M ± SD		CL ^3^ (LL, UL)		Cohen’s d_z_
RA (% correct)	Pre-meditation	91.4	4.2	89.5	93.4	1.117
	Post-meditation	90.1	6.7	87.0	93.2	
RT (ms)	Pre-meditation	824.6	127.1	764.1	883.1	0.324
	Post-meditation	735.8	98.2	689.8	781.7	
MAP (mmHg)	Pre-meditation	86.4	6.6	82.3	90.5	0.053
	Post-meditation	86.1	7.7	81.3	90.9	
PP (mmHg)	Pre-meditation	44.8	9.1	39.2	50.4	0.827
	Post-meditation	39.6	9.2	33.9	45.3	
HR (bpm)	Pre-meditation	80.6	12.7	71.3	89.7	0.417
	Post-meditation	77.9	11.7	69.5	86.3	

KEY: M—mean; SD—standard deviation; ^1^ Behavioral measures (*n* = 20); ^2^ Physiological measures (*n* = 10); ^3^ Confidence Intervals (95%) with the lower limit (LL) and upper limit (UL) for each condition and variable. Cohen’s d_z_ effect size index is noted for each variable [29].

**Table 2:** Descriptive statistics for the fNIRS measures for each channel separately across the Stroop test before meditation, after meditation, and Stroop test after meditation conditions.

		SCWT-Pre		Meditation		SCWT-Post	
Dependent Variable	Channel	M ± SD		M ± SD		M ± SD	
HbO_2_ (μMol)	1	0.715	1.355	1.201	2.532	0.553	1.653
	2	0.197	1.251	0.280	1.853	0.155	1.607
	3	0.803	1.391	1.830	2.085	0.987	2.108
	4	0.309	1.262	0.092	2.089	0.368	1.752
Hb (μMol)	1	−0.421	0.544	0.050	0.660	0.219	0.533
	2	−0.568	0.780	0.089	0.793	−0.317	0.474
	3	−0.488	0.720	−0.073	1.122	0.239	0.888
	4	−0.366	0.764	−0.104	0.853	−0.420	0.676
HbT (μMol)	1	0.294	1.152	1.252	2.666	0.334	0.885
	2	−0.271	1.210	0.369	1.893	−0.162	0506
	3	0.315	1.505	1.757	2.240	0.748	2.468
	4	−0.057	0.994	−0.012	1.438	−0.523	1.877

KEY: M—mean; SD—standard deviation; SCWT-pre—Stroop test before meditation; SCWT-post—Stroop test after meditation; HbO_2_—oxygenated hemoglobin; Hb—deoxygenated hemoglobin; Hb—total hemoglobin; μMol—mean micromolar units.

**Table 3:** Separate linear regressions of meditation effects with hemodynamic measures obtained by fNIRS (*n* = 20), physiological measures (*n* = 10), and pre-meditation Stroop behavioral measures on the Stroop behavioral measures post-meditation.

Response Variable	Predictor	USC Beta	SE	SC Beta	*t*-Value	*p*	*R* ^2^	Adj *R^2^*
RA-SCWT-post	MedeffHbO_2_	−0.741	0.186	−0.257	−3.975	<0.001	0.716	0.707
	RA−SCWT−pre	1.242	0.101	0.796	12.306	<0.001		
	MedeffHb	0.495	0.422	0.089	1.173	0.245	0.656	0.646
	RA−SCWT−pre	1.305	0.117	0.837	11.030	<0.001		
	MedeffHbT	−0.732	0.206	−0.241	−3.558	<0.001	0.705	0.696
	RA−SCWT−pre	1.170	0.106	0.750	11.077	<0.001		
	MedeffHR	0.315	0.090	0.268	3.482	0.001	0.748	0.737
	RA−SCWT−pre	1.521	0.132	0.886	11.533	<0.001		
	MedeffPP	−0.259	0.059	−0.309	−4.367	<0.001	0.776	0.766
	RA−SCWT−pre	1.398	0.121	0.815	11.530	<0.001		
	MedeffMAP	0.288	0.095	0.236	3.033	0.004	0.735	0.723
	RA−SCWT−pre	1.351	0.133	0.787	10.124	<0.001		
RT-SCWT-post	MedeffHbO_2_	−0.617	3.381	−0.014	−0.183	0.856	0.615	0.604
	RT−SCWT−pre	0.624	0.060	0.784	10.421	<0.001		
	MedeffHb	−5.053	7.123	−0.058	−0.709	0.481	0.618	0.607
	RT−SCWT−pre	0.605	0.065	0.761	9.267	<0.001		
	MedeffHbT	−2.064	3.666	−0.044	−0.563	0.575	0.617	0.616
	RT−SCWT−pre	0.616	0.062	0.774	9.999	<0.001		
	MedeffHR	−2.164	1.337	−0.125	−1.619	0.112	0.744	0.733
	RT−SCWT−pre	0.664	0.062	0.829	10.775	<0.001		
	MedeffPP	0.016	1.001	0.001	0.016	0.987	0.729	0.727
	RT−SCWT−pre	0.685	0.065	0.854	10.577	<0.001		
	MedeffMAP	2.584	1.674	0.143	1.543	0.130	0.743	0.742
	RT−SCWT−pre	0.618	0.074	0.771	8.305	<0.001		

KEY: USC Beta—unstandardized regression coefficients; SE—standard error of the regression coefficients; SC Beta—standardized regression coefficients; t-value—two-sided observed significance values for the t-statistics; *p*—significance values; *R*^2^- squared multiple correlation coefficient; Adjusted *R*^2-^—adjusted multiple correlation coefficient; RA-SCWT-pre—response accuracy Stroop pretest score (%); RA-SCWT-post—response accuracy Stroop posttest score (%); RT-SCWT-pre—response time Stroop pretest score (msec); RT-SCWT-post—response time Stroop post test score (msec); SCWT-pre = Stroop test before meditation; SCWT-post = Stroop test after meditation; HbO_2_-oxygenated hemoglobin; Hb-deoxygenated hemoglobin; HbT-total hemoglobin; μMol-mean micromolar units; MAP—mean arterial pressure (mmHg); PP—pulse pressure (mmHg); HR -heart rate (bpm); Medeff—meditation effect; for each meditation effect the value of either the pretest for the physiological or hemodynamic biomarker was subtracted from the meditation effect, e.g., for MedeffHb = MedHb − PreHb.

**Table 4:** Descriptive statistics and separate one-sample *t*-tests for fNIRS laterality measures for the Stroop test before meditation, after meditation, and Stroop test after meditation conditions.

Dependent Variable	*t*	df	*p*	Difference	Cohen’s d	Mean	SD
LIPreHbO_2_	−1.160	18	0.261	−0.097	−0.266	−0.097	0.365
LIMedHbO_2_	−1.913	18	0.072	−0.146	−0.439	−0.146	0.332
LIPstHbO_2_	0.132	19	0.897	0.017	0.029	0.017	0.593
LIPreHb	−0.222	18	0.827	−0.029	−0.051	−0.029	0.574
LIMedHb	−0.052	18	0.959	−0.003	−0.012	−0.003	0.238
LIPstHb	3.649	18	0.002	0.522	0.837	0.522	0.623

KEY: M-mean; SD-standard deviation; *t*—one-sample *t*-test with difference from 0; df—degrees of freedom; *p*—*p*-value; Cohen’s d—standardized mean difference effect size index; LI—laterality index; Pre-Stroop test before meditation; Post-Stroop test after meditation; HbO_2_-oxygenated hemoglobin; Hb-deoxygenated hemoglobin; All measures are in μMol-mean micromolar units.
